# Retinal Challenges in Sickle Cell Patients—Present and Future: A Systematic Review

**DOI:** 10.3390/bioengineering13080867

**Published:** 2026-07-27

**Authors:** Elie Motulsky, Amina El Bachti

**Affiliations:** Department of Ophthalmology, Erasme Hospital, Hôpital Universitaire de Bruxelles (H.U.B.), Université Libre de Bruxelles (U.L.B.), 1070 Brussels, Belgium

**Keywords:** retina, sickle cell disease, retinopathy, maculopathy, retinal complications, risk factors, screening, ophthalmic follow-up

## Abstract

**Importance:** Sickle cell retinopathy and maculopathy are frequent but underdiagnosed complications of sickle cell disease that can lead to vision loss. Unfortunately, there is currently no ophthalmological follow-up protocol to best diagnose and treat these complications. **Objective:** To identify the most important risk factors associated with the progression of sickle cell retinopathy and maculopathy, review the current screening techniques and effective treatments and propose a follow-up protocol tailored to the patient’s risk profile. **Evidence Review:** Relevant studies were selected using the PICO framework and a structured search strategy across multiple databases including PubMed, Scopus, Cochrane Library, Cible+, ScienceDirect and the American Academy of Ophthalmology network. The search spanned from 1980 to 2025. Original research articles addressing risk factors, screening, and treatment methods for sickle cell retinopathy and maculopathy were included. In total, 48 studies met the inclusion criteria. Data extraction and quality assessment were applied uniformly. **Findings:** The systematic review included 48 articles (10,543 participants), representing a mix of study types: randomized clinical trials, prospective and retrospective cohort studies, and cross-sectional studies. Major findings included: **Risk Factors**: Commonly identified risk factors for sickle cell retinopathy and maculopathy include genotype SS, increased age, male sex, and low fetal hemoglobin levels. **Screening and Diagnosis**: Wide-field fluorescein angiography and optical coherence tomography angiography were the most effective tools for early detection of peripheral ischemia and macular thinning, respectively. **Treatment and Management**: Laser photocoagulation remains the standard intervention for proliferative sickle cell retinopathy. Preventive strategies and regular ophthalmologic monitoring are emphasized across high-quality studies. **Conclusions and Relevance:** Risk stratification based on genotype, hemoglobin F levels, and imaging findings allows for tailored ophthalmologic management of patients with sickle cell disease. A structured follow-up protocol is proposed, aiming to improve early detection, prevent complications, and optimize resource allocation.

## 1. Introduction

Sickle cell disease (SCD) is the most common genetic hemoglobinopathy worldwide and represents a growing public health concern due to increasing life expectancy and migratory patterns [1,2]. In Belgium, for example, the incidence has increased significantly, with approximately 1 in 2000 children now affected [3]. SCD results from a point mutation in the β-globin gene, leading to hemoglobin S through normal hemoglobin A polymerization under hypoxia and causing vaso-occlusion, hemolysis, and systemic inflammation [4,5,6,7]. The homozygous form SS (HbSS) and compound heterozygous variants like HbSC and HbS/thalassemia are associated with more severe clinical manifestations [4]. SCD leads to multisystemic complications, including ophthalmologic manifestations that are often underrecognized despite their potential to cause irreversible vision loss [8].

Ocular manifestations affect approximately 30–50% of adults with sickle cell disease [8]. The most frequent ones include sickle cell retinopathy (SCR) and sickle cell maculopathy (SCM) [8]. SCR arises from peripheral retinal ischemia due to vaso-occlusion and is staged according to the Goldberg classification [9,10,11]. SCM affects the temporal macula and is associated with capillary dropout and thinning of the inner retinal layers, which may lead to paracentral scotomas [12,13]. Despite their potential severity, these complications frequently progress silently and are often detected late.

Progressive retinal ischemia may result in vitreous hemorrhage, retinal detachment, permanent visual impairment, reduced reading ability, loss of independence, and diminished vision-related quality of life [8]. Beyond visual disability, repeated ophthalmologic visits, invasive angiography, and treatment procedures contribute additional physical and psychological burden in patients who already require lifelong multidisciplinary care.

There is currently no international consensus on standardized ophthalmologic screening protocols for patients with SCD. Screening intervals and modalities vary widely across institutions, which contributes to inconsistent early detection and delayed intervention [14]. Current management generally consists of periodic dilated retinal examination supplemented by ultra-wide-field imaging when available. Laser photocoagulation remains the standard treatment for proliferative sickle cell retinopathy, whereas non-proliferative disease is generally monitored with regular follow-up [12,13,14].

Given the heterogeneity in screening recommendations, the absence of validated risk stratification tools, and the evolving evidence base for diagnostic imaging and treatment, there is a clear need to synthesize current evidence systematically.

The objectives of this systematic review are to:

1. Identify and synthesize evidence on risk factors associated with the development and progression of SCR and SCM.
2. Evaluate the diagnostic accuracy and clinical utility of current screening modalities.
3. Review the evidence for treatment interventions.
4. Propose a risk-adapted, evidence-based protocol for ophthalmologic surveillance in SCD, integrating patient-specific factors and imaging findings.

By collating evidence from diverse geographic and healthcare settings, this review aims to provide clinicians with an actionable framework for individualized patient management and to identify critical gaps warranting future research.

## 2. Methods

### 2.1. Protocol and Reporting Guidelines

**Figure 1:**
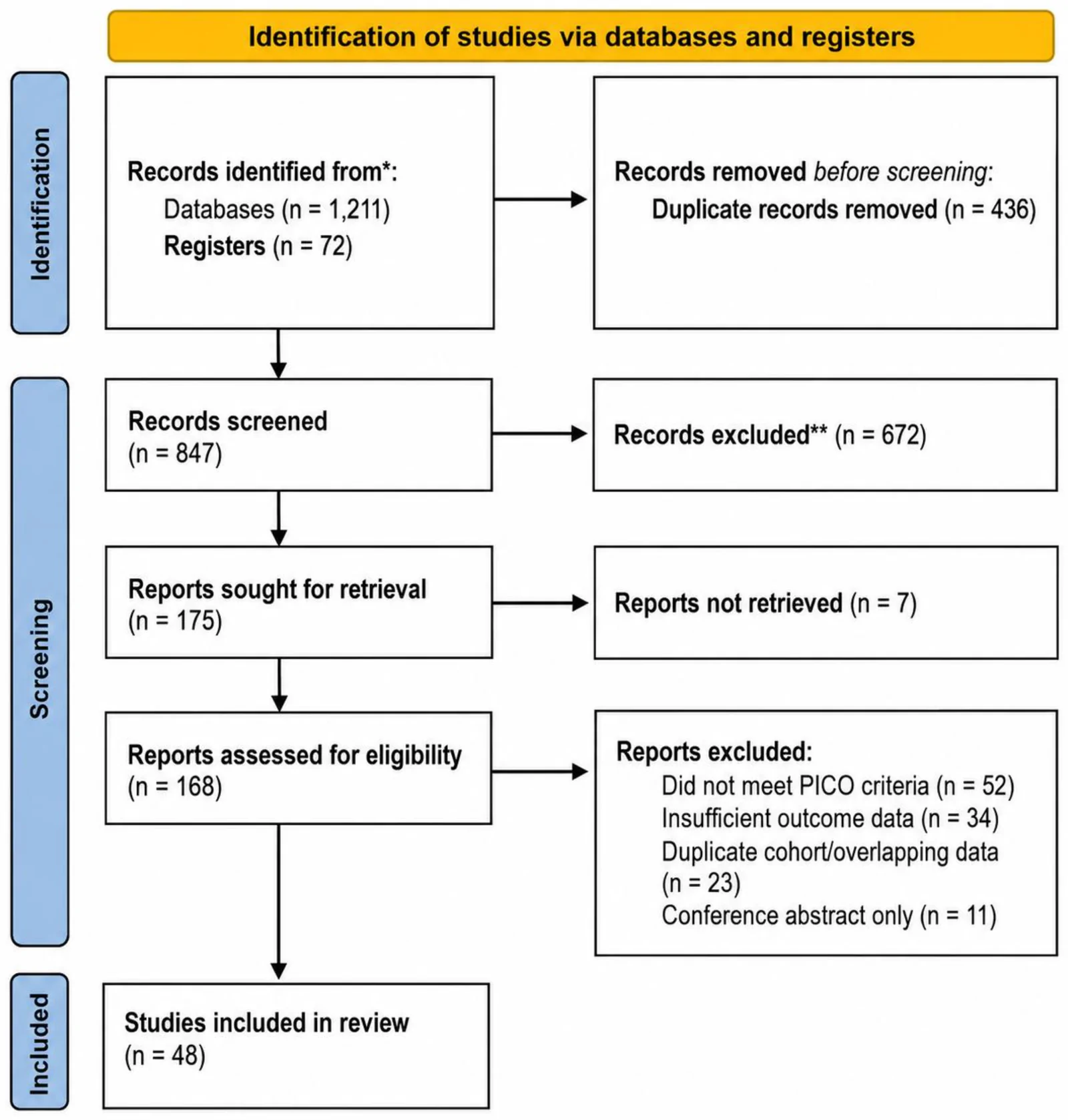
Our PRISMA flow diagram. *: PubMed/MEDLINE; Scopus; Cochrane Central Register of Controlled Trials; ScienceDirect; Cible+. **: not relevant to SCR/SCM; review articles/editorials; case reports (<5 patients); animal/in vitro studies; non-English/French.

This systematic review was conducted in accordance with the Preferred Reporting Items for Systematic Reviews and Meta-Analyses (PRISMA) 2020 statement. To illustrate the selection process, a PRISMA flow diagram was created using Microsoft Word for Microsoft 365 (Microsoft Corporation, Redmond, WA, USA) (Figure 1). The protocol was developed prospectively but was not registered in PROSPERO or another systematic review registry. A completed PRISMA 2020 checklist is provided in Supplementary Material Table S1.

### 2.2. Eligibility Criteria

Studies were eligible if they:

- Were original peer-reviewed research articles;
- Were published in English or French;
- Were published between 1 January 1980 and 30 April 2025;
- Included patients with sickle cell disease;
- Evaluated sickle cell retinopathy and/or sickle cell maculopathy;
- Investigated at least one of the following:
  ♦ Risk factors;
  ♦ Screening;
  ♦ Imaging;
  ♦ Treatment;
  ♦ Prognosis.

Exclusion criteria included:

- Narrative reviews, editorials, conference abstracts, letters to the editor, case reports (fewer than 5 patients), and study protocols;
- Animal or in vitro studies;
- Studies not available in English or French;
- Studies focusing exclusively on non-retinal ocular manifestations (e.g., anterior segment findings only).

### 2.3. Information Sources

A systematic search was performed across the following databases:

- PubMed/MEDLINE;
- Scopus;
- Cochrane Central Register of Controlled Trials;
- ScienceDirect;
- Cible+ (French biomedical database).

Supplementary searches were conducted through the American Academy of Ophthalmology (AAO) publication network. Additional studies were identified by screening reference lists of included articles and relevant systematic reviews and by using PubMed “Similar Articles” and “Cited by” functions.

The search was conducted between 1 February and 30 April 2025.

### 2.4. Search Strategy

The search strategy was structured according to the PICO framework and combined controlled vocabulary and free-text terms related to sickle cell disease (“sickle cell disease”, “sickle cell anemia”, “hemoglobin S disease”), ocular manifestations (“retinopathy”, “sickle cell retinopathy”, “maculopathy”), risk factors (“risk factors”), and management (“screening”, “treatment”, “management”, “monitoring”, “best practice”).

♦ Population

Patients of any age with sickle cell disease (HbSS, HbSC, HbS/β-thalassemia, or other compound heterozygous variants).

♦ Intervention

*Screening and Diagnostic Modalities:*

Dilated fundus examination;

Fundus photography;

Wide-field fluorescein angiography (WFA);

Ultra-wide-field fluorescein angiography (UWFA);

Ultra-wide-field fundus photography (UWFP);

Spectral-domain optical coherence tomography (SD-OCT);

Optical coherence tomography angiography (OCTA).

*Therapeutic Interventions:*

Laser photocoagulation (sectoral, scatter, feeder vessel);

Anti-vascular endothelial growth factor (anti-VEGF) intravitreal injection;

Pars plana vitrectomy;

Scleral buckling;

Hydroxyurea (systemic therapy with potential ocular effects);

Blood transfusion (systemic therapy with potential ocular effects).

*Risk Factor Exposures:*

Genotype (HbSS, HbSC, HbS/β-thalassemia);

Fetal hemoglobin (HbF) levels;

Hematological parameters (hemoglobin, hematocrit, mean corpuscular volume);

Age, sex, and other demographic factors;

Systemic comorbidities.

♦ Comparators

Alternative screening modality or no screening;

Alternative treatment or observation;

Absence of risk factor exposure.

♦ Outcomes

*Primary*

Prevalence;

Progression;

Proliferative SCR;

SCM;

Sensitivity.

*Secondary*

Visual outcomes;

Treatment success;

Complications;

Follow-up recommendations.

### 2.5. Study Selection

Records identified through database searching were imported and screened for duplication. Titles and abstracts were screened for relevance. Potentially eligible studies underwent full-text review according to the predefined eligibility criteria. Additional articles identified through citation searching were assessed using the same process.

### 2.6. Data Extraction

Data were extracted independently by two reviewers (Elie Henri Motulsky (E.H.M.) and Amina El Bachti (A.E.B.)) using a standardized data extraction form piloted on five studies before full implementation. Extracted data included:

- Study characteristics: author, year, country, study design, sample size, follow-up duration;
- Participant characteristics: age (mean, range), sex distribution, genotype distribution;
- Exposure/intervention details: screening modality, treatment type and protocol;
- Outcome measures: prevalence/incidence of retinopathy/maculopathy, diagnostic accuracy metrics, treatment efficacy, visual acuity, complications;
- Risk factor associations: effect estimates with confidence intervals where reported.

Discrepancies in data extraction were resolved by comparison of extraction forms and consensus discussion. For studies with missing or unclear data, corresponding authors were contacted when feasible.

### 2.7. Quality Assessment and Risk of Bias

*Observational studies and cross-sectional studies*: The Newcastle–Ottawa Scale (NOS) was used for cohort and case–control studies. The NOS evaluates three domains: selection of study groups, comparability of groups, and ascertainment of outcome/exposure. Study scores were classified as low risk of bias, moderate risk, and high risk.

*Randomized controlled trials:* The Cochrane Risk of Bias 2 (RoB 2) tool was used, assessing five domains: bias arising from the randomization process, bias due to deviations from intended interventions, bias due to missing outcome data, bias in measurement of the outcome, and bias in selection of reported results Each domain was judged as “low risk,” “some concerns,” or “high risk,” with an overall judgment derived according to the RoB 2 algorithm.

Domain-level and overall risk-of-bias assessments are reported in Supplementary Material Table S2.

Methodological reporting quality was descriptively assessed using design-specific reporting guidelines for descriptive purposes: CONSORT for randomized trials, STROBE for observational studies and TREND for non-randomized intervention studies. These assessments were not used as exclusion criteria. These assessments were used to evaluate the methodological robustness of the available studies but were not used as criteria for study exclusion or quantitative weighting in the synthesis.

Study selection, data extraction and quality assessment were performed by a single reviewer using predefined eligibility criteria and standardized extraction forms. Although this ensured consistency, the absence of an independent second reviewer may have introduced selection bias and is acknowledged as a limitation.

### 2.8. Data Synthesis

Meta-analysis was not appropriate because of substantial heterogeneity regarding study design, imaging modalities, outcome definitions, follow-up duration, and patient characteristics. Findings were therefore synthesized narratively. For variables consistently reported across studies, weighted arithmetic means were calculated by weighting individual study estimates according to their sample sizes. Weighted arithmetic means were calculated only for descriptive variables consistently reported across studies. These summaries were intended solely to facilitate descriptive comparison and should not be interpreted as pooled effect estimates.

Formula:

$${\bar{x}}_{weighted}=\;\frac{\sum nixi}{\sum ni}$$

where:

- xi = reported value;
- ni = sample size.

Sample-size weighting was chosen to ensure that estimates from larger studies contributed proportionally more to the descriptive summary than those from smaller studies. These weighted means were intended solely to provide descriptive summaries of the available literature and should not be interpreted as pooled effect estimates from meta-analysis or measures of treatment efficacy.

Data were organized and analyzed using Microsoft Excel for Microsoft 365 (Microsoft Corporation, Redmond, WA, USA). Results are structured by outcome category: clinical outcomes (prevalence, progression, visual acuity), diagnostic outcomes (sensitivity, specificity of screening modalities), and therapeutic outcomes (treatment efficacy and complications).

To illustrate the selection process, a PRISMA flow diagram was created (Figure 1).

## 3. Results

### 3.1. Overview of Included Studies

A total of 48 studies met inclusion criteria and were included in the qualitative synthesis. The included studies comprised 3 randomized controlled trials, 24 prospective observational studies, and 21 retrospective observational studies, representing a total of 10,543 patients with SCD. Studies originated from 15 countries across 5 continents, with the largest contributions from the United States (*n* = 16), France (*n* = 7), Jamaica (*n* = 4), and the United Kingdom (*n* = 4). Collectively, these studies cumulated data from 10,543 patients living with SCD, with diverse genotypes and clinical presentations. Follow-up duration ranged from cross-sectional assessment to 20 years of longitudinal observation. Study characteristics are detailed in Table 1.

### 3.2. Risk-of-Bias Assessment

*Observational Studies*

Among the 45 observational studies assessed using the NOS, 15 were classified as low risk of bias, 25 as moderate risk, and 5 as high risk.

The most common methodological limitations were in the comparability domain, where 51% of studies did not adequately control key confounders such as fetal hemoglobin levels, hydroxyurea use, or transfusion history. Additionally, 33% of studies had inadequate follow-up duration (<2 years) or significant loss to follow-up (>20%).

*Randomized Controlled Trials*

The three RCTs were assessed using the Cochrane RoB 2 tool. One trial (Sayag et al.) was judged as low risk of bias overall, with adequate randomization, allocation concealment, and complete outcome reporting. Two trials (Jampol et al., Farber et al.) raised some concerns, primarily due to incomplete description of randomization procedures, open-label design, and potential performance bias.

### 3.3. Risk Factors

#### 3.3.1. Sickle Cell Retinopathy

##### Genotype for SCR

**Figure 2:**
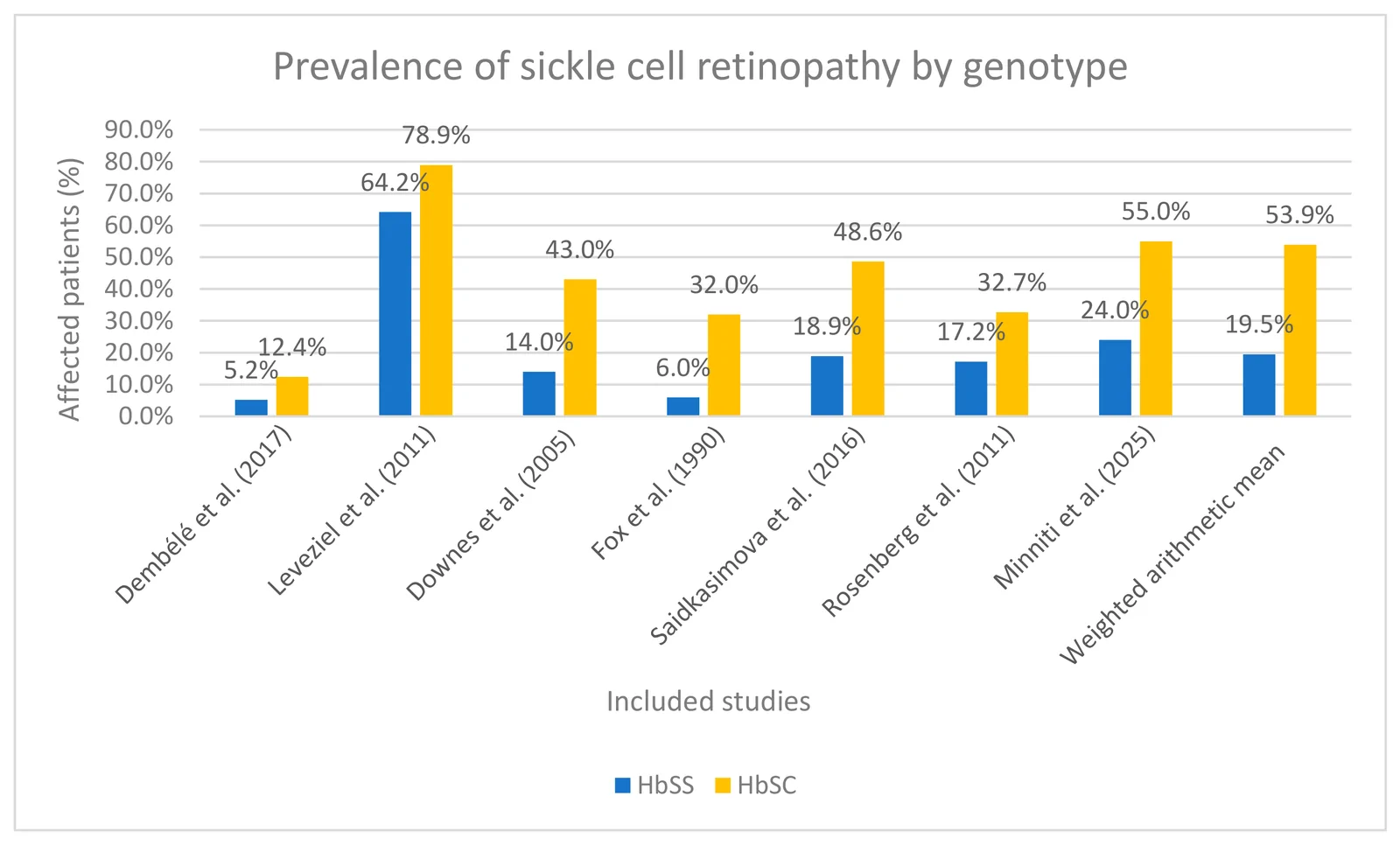
Prevalence of proliferative sickle cell retinopathy by genotype [17,18,19,20,21,22,23]. The weighted arithmetic mean was calculated from the prevalence values reported in the included studies. HbSC: hemoglobin SC, HHbSS: hemoglobin SS.

The genotype plays a central role in determining SCR risk [15,16]. The HbSC genotype is consistently associated with a higher incidence of proliferative SCR compared with the HbSS (Figure 2 [17,18,19,20,21,22,23]. Figure 2 clearly shows that across all the studies collected, the prevalence of SCR based on genotype is higher for HbSC patients. One article suggests that HbSC patients exhibit distinct endothelial gene expression patterns that may promote retinal angiogenesis and contribute to PSCR development [24]. 

##### Hemorheological Parameters

Hemorheological parameters, including blood viscosity, are also significant contributors [25]. However, a recent study found no association between blood viscosity and SCR, suggesting alternative mechanisms may be involved [26]. Elevated hemoglobin (>12 g/dL) and hematocrit levels have been independently associated with increased SCR prevalence [17,26,27,28].

##### Age

**Figure 3:**
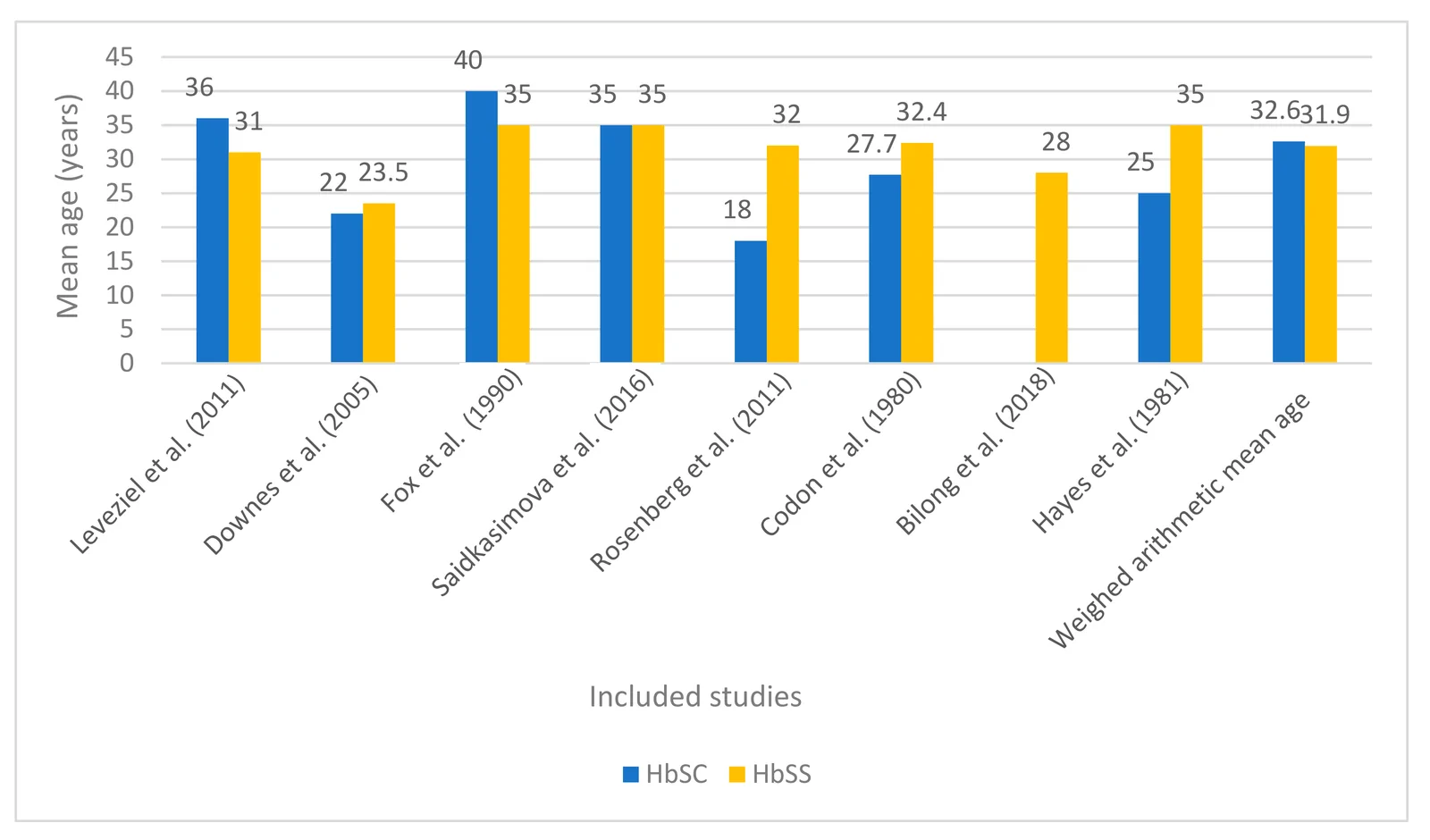
Average age associated with the risk of developing sickle cell retinopathy by genotype [18,19,20,21,22,30,31,32]. The weighted arithmetic mean was calculated from the mean age reported in the included studies HbSC: hemoglobin SC, HbSS: hemoglobin SS.

Age is a strong predictive risk factor for SCR [29], with prevalence increasing steadily across the lifespan and reaching up to 80.5% by age 40 (Figure 3) [18,19,20,21,22,30,31,32].

##### Male Sex

Male sex has also been associated with an increased risk of progression to proliferative forms [29]. This observation aligns with the gender ratios reported in recent studies: Hayes et al. and Fox et al. [20,32] both found a ratio of 1.5, while Downes et al. [19] reported a slightly higher ratio of 1.7. Rosenberg et al. [22] observed an even higher ratio of 2.3. In contrast, Dembélé et al. [17] reported a lower ratio of 0.8. The weighted arithmetic sex ratio across these studies was 1.3, further supporting the trend of male predominance in disease progression.

##### Fetal Hemoglobin Levels

Fetal hemoglobin (HbF) levels inversely correlate with SCR risk, as higher HbF concentrations reduce HbS polymerization and exert a protective effect, with statistically significant associations observed in both pediatric and adult populations [27,33]. This relationship is supported by clinical studies reporting varying HbF levels: Dembélé et al. [17] documented 15% HbF, while Estepp et al. [34] observed 20% HbF, and Brandsen et al. [27] reported 15% HbF. The weighted arithmetic mean across these studies was 17.7%, reinforcing the potential role of elevated HbF in mitigating SCR progression.

##### Systemic and Hematological Factors for SCR

Key risk factors include high hemoglobin and hematocrit levels [31], high mean corpuscular volume (MCV), tobacco use, elevated leukocyte levels, hematuria, osteonecrosis, and a history of gallstones, cholecystitis, or cholecystectomy [17,34]. Conversely, protective factors include hydroxyurea therapy [17,18,33,34,35] and previous transfusions [18]. All reported associations are statistically significant (*p* < 0.05).

#### 3.3.2. Sickle Cell Maculopathy

##### Genotype for SCM

In contrast to SCR, the SS genotype is more strongly associated with the development of SCM [36,37]. Studies by Mathew et al. [38] reported a prevalence of 55.9% for HbSS and 13.50% for HbSC, while Orssaud et al. [39] documented 53.4% for HbSS and 23.70% for HbSC. The weighted arithmetic mean across included studies was 48% for HbSS and 35% for HbSC, reinforcing the higher burden of maculopathy in HbSS genotypes.

##### Prothrombin Time

Fares et al. hypothesized that prolonged prothrombin time (PT)—reflected by lower prothrombin levels—is associated with chronic vascular inflammation and an increased risk of SCM (*p* = 0.01). A threshold of PT < 79.2% has been proposed as a risk marker for SCM [36].

##### Systemic and Hematological Factors for SCM

Hemolysis appears to play a central role in SCM development. Decreased hemoglobin and hematocrit levels, alongside elevated lactate dehydrogenase (LDH), have been linked to the disease [36]. In contrast, elevated HbF levels may exert a protective effect [40]. Moreover, systemic comorbidities such as hypertension, acute chest syndrome, and diabetes have been associated with more severe manifestations of SCM [37].

##### Protective Systemic Treatments

Previous transfusions may offer protection against SCM by reducing hemolytic activity. Similarly, hydroxyurea, through its ability to increase HbF levels, has been identified as a protective therapy [40].

### 3.4. Screening Techniques

Several studies recommend initiating SCR screening at 9 years of age for patients with the SC genotype and at 13 years of age for those with the SS genotype [41,42]. Screening is generally recommended every 2 years if no retinopathy is detected and annually once SCR is present [43].

#### 3.4.1. Fundus Examination

The AAO and the National Heart, Lung, and Blood Institute recommend screening every 1 to 2 years if the fundus examination is normal 63. While fundus examination (FE) remains the standard initial clinical assessment, its sensitivity to detect abnormalities is limited [44].

#### 3.4.2. Ultra-Wide-Field Fundus Photography

Ultra-wide-field fundus photography (UWFP) shows greater sensitivity than FE for detecting peripheral retinal capillary occlusion [45].

#### 3.4.3. Wide-Field Fluorescein Angiography and Ultra-Wide-Field Fluorescein Angiography

**Figure 4:**
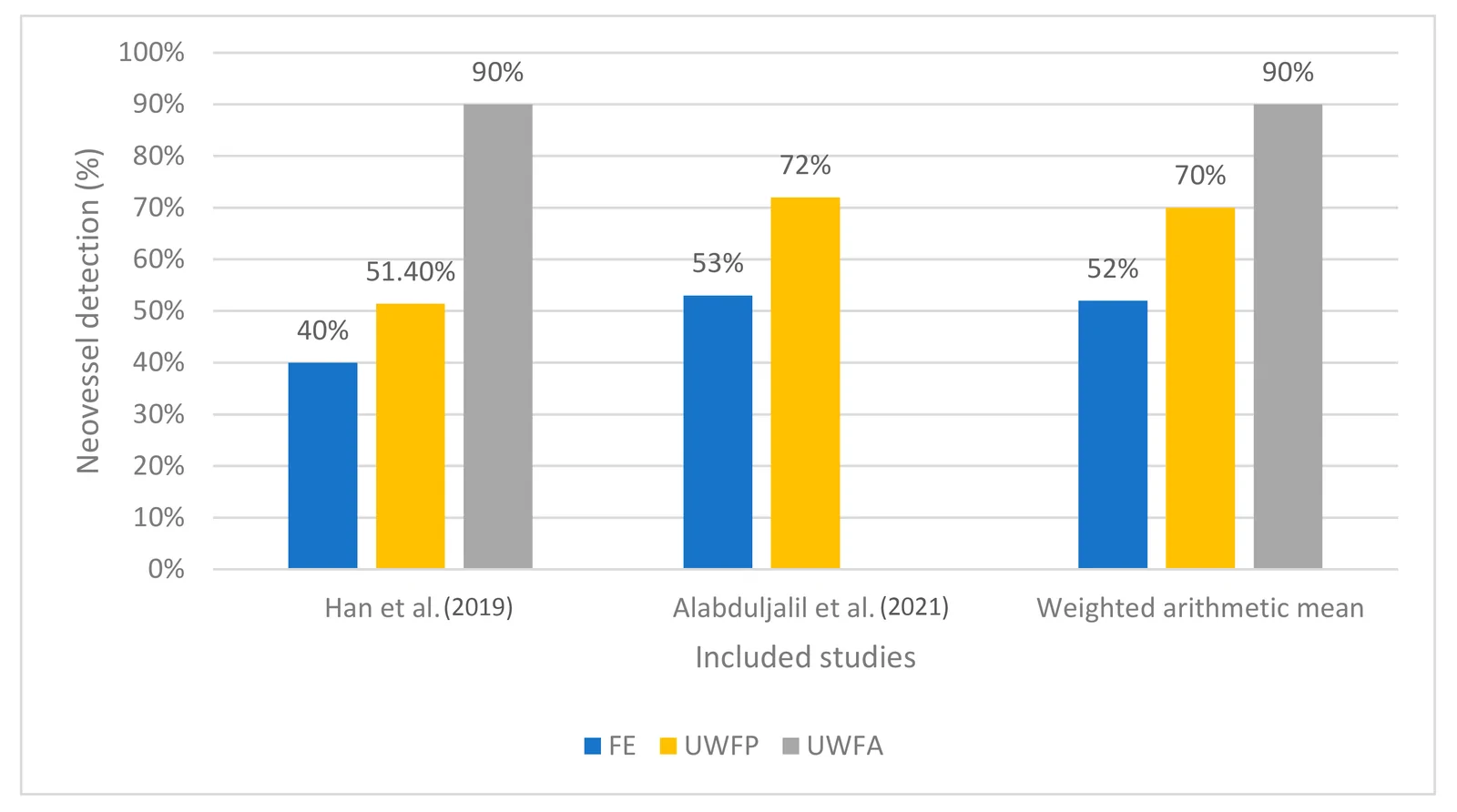
Sensitivity of different screening techniques to detect sickle cell retinopathy [44,45]. The weighted arithmetic mean was calculated from the sensitivity of neovessel detection values reported in the included studies FE: fundus examination, UWFP: ultra-wide-field fundus photography, UWFA: ultra-wide-field fluorescein angiography.

Wide-field fluorescein angiography (WFA) (≥100° field of view) is considered the gold standard for SCR staging and follow-up [46]. Ultra-wide-field fluorescein angiography (UWFA), with a field of view of up to 200°, detects peripheral vascular anomalies in 98.6% of cases [47]. In a study by Han et al., UWFA demonstrated 90% sensitivity in detecting retinal lesions, outperforming both FE and UWFP (Figure 4) [44,45,48].

#### 3.4.4. Spectral-Domain Optical Coherence Tomography and OCT Angiography

Spectral-domain optical coherence tomography (SD-OCT) enables detailed analysis of the macula [49,50], while OCT Angiography (OCTA) offers non-invasive, qualitative and quantitative assessment of central retinal microvasculature [36,37,51,52]. A study by Minvielle et al. showed that OCTA detected twice as many perifoveal microvascular abnormalities as WFA [53].

### 3.5. Treatment

#### 3.5.1. Systemic and Preventive Treatments

Hydroxyurea remains the primary systemic therapy for sickle cell disease, increasing HbF levels and reducing vaso-occlusive events. Its use has been associated with a lower incidence of SCR, reported at 8% in treated patients compared with 14% in untreated individuals [34]. Blood transfusions also offer protection against ocular complications by reducing hemolysis and improving oxygen delivery.

#### 3.5.2. Observational Approach

**Figure 5:**
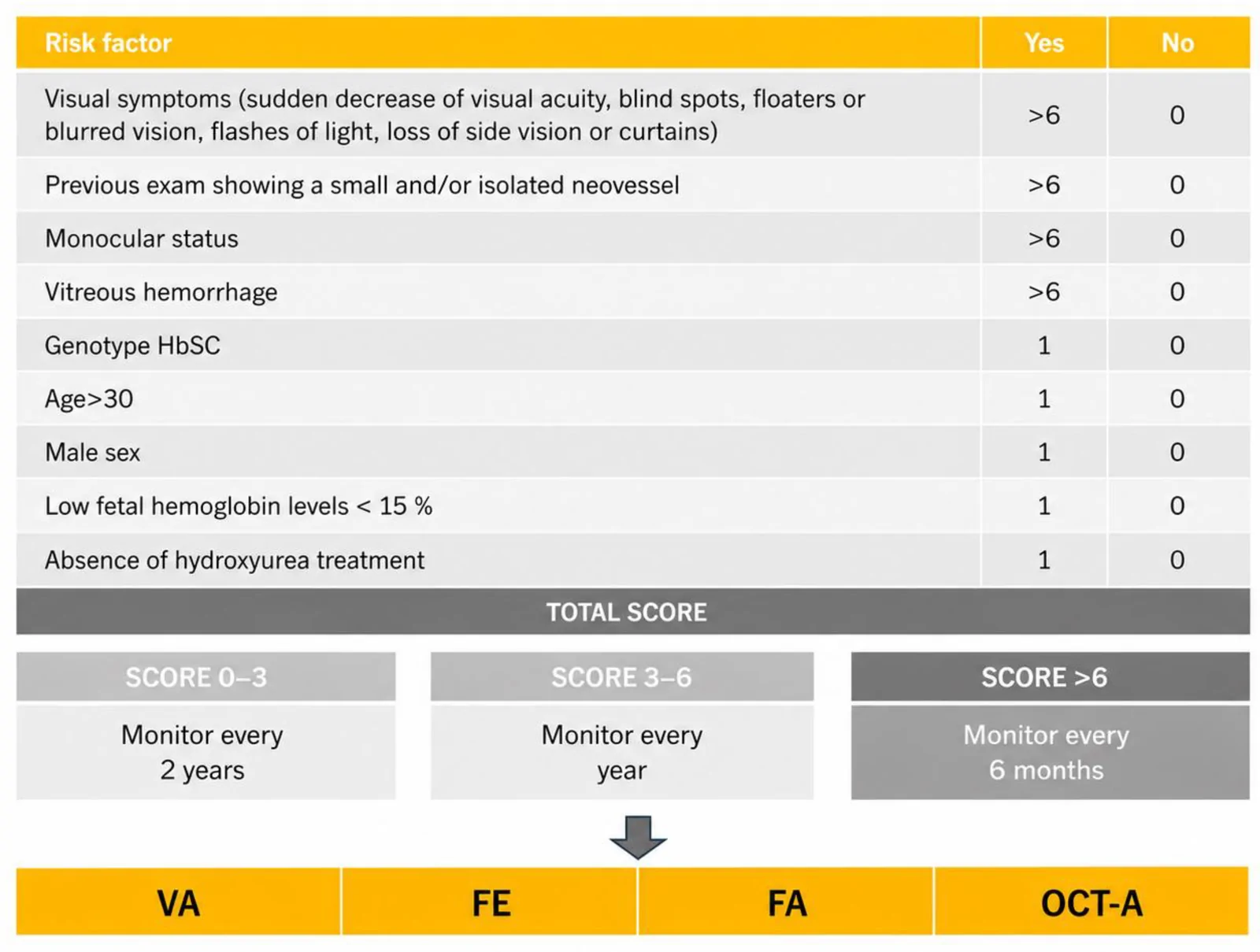
Recommended management of sickle cell retinopathy. ***HbSC:*** *hemoglobin SC. Author-proposed algorithm derived from literature synthesis.*

Proliferative SCR demonstrates variable rates of spontaneous regression of neovascularization (autoinfarction), reported in 32% of cases by Downes et al. [19] and 61% by Codon et al. [30]; the weighted arithmetic mean across these studies was 34.7%, reinforcing the observational approach. It may be considered in cases with limited neovascularization and no vitreous hemorrhage. However, regular follow-up remains essential to avoid further complications. In this context, we propose a risk score (Figure 5) integrating seven clinical and biological factors to stratify patients into low-, intermediate-, and high-risk categories. This score helps guide follow-up intervals—ranging from every 6 months to every 2 years—and ensures appropriate timing of intervention in line with disease activity and patient-specific risk.

#### 3.5.3. Ophthalmic Interventions

##### Laser Photocoagulation

Randomized clinical trials have demonstrated that feeder vessel photocoagulation provides greater protection against vitreous hemorrhage (VH) and vision loss compared with sectoral photocoagulation [54,55]. However, the technique was associated with a higher incidence of complications, including retinal detachment (RD), and is no longer routinely recommended. Current guidelines favor targeted sectorial panretinal photocoagulation directed toward ischemic retinal zones located anteriorly to the neovascular lesions [56].

##### Anti-Vascular Endothelial Growth Factor Injections

Intravitreal injections of anti-vascular endothelial growth factor (anti-VEGF) agents, such as bevacizumab, have been shown to reduce neovascularization and VH in patients with proliferative SCR [57]. These agents are commonly used as adjuncts to laser therapy or preoperatively prior to vitrectomy. However, the optimal dosing intervals and long-term efficacy remain unclear, as neovascularization may recur after treatment [58].

##### Pars Plana Vitrectomy

Pars plana vitrectomy (PPV) is reserved for complications such as non-resolving VH, RD, macular hole, or symptomatic tractional membranes [59]. Long-term data from a 12-year follow-up study suggest that PPV improves visual acuity, particularly in eyes with VH or epiretinal membranes [60]. Nonetheless, a recurrence rate of approximately 50% of RD post-surgery has been reported [59,61]. A recent study showed that scleral buckling combined with pars plana vitrectomy achieved slightly higher rates of single-operation anatomic success compared with PPV alone, although final visual acuity outcomes were similar in the two groups [62].

## 4. Discussion

SCD is a debilitating condition responsible for multiple comorbidities, often leading to frequent and prolonged hospitalization periods [64]. These patients, often from an early age, must cope with multiple hospital admissions and numerous medical follow-ups [65]. Consequently, ophthalmological care of SCD patients is an additional burden and stress to their already complex health needs [66].

This systematic review proposes distinct risk stratification tables for SCR and SCM development (Table 2), categorizing factors as high, moderate, or low likelihood based on their weighted importance across studies. This tiered classification enables clinicians to prioritize monitoring for patients with multiple high-risk factors while avoiding over-surveillance of those with unlikely markers.

Many studies examined in this review used WFA as a first-line procedure or in cases of doubt due to its greater sensitivity in detecting peripheral retinal lesions. However, this test is often poorly tolerated, especially in patients with difficult venous access [59,67]. Similarly, interventions such as laser photocoagulation, or more rarely, intravitreal anti-VEGF injections, are also often poorly tolerated and can lead to side effects. It is therefore essential to assess the overall impact of this treatment on patients’ quality of life and to consider the risk–benefit balance when considering the indications for screening and treatment of SCR [68,69]. These factors underscore the need to balance clinical efficacy with quality-of-life considerations.

Despite the frequent use of non-invasive examinations and less burdensome tests, all studies have concluded that WFA has demonstrated its superiority over FE and Wide-Field photographs [53,70]. Available evidence consistently identifies WFA as the preferred imaging modality for detecting peripheral retinal ischemia and monitoring its progression and response to treatment, although this conclusion is primarily supported by observational studies. Concerning macular damage in SCD, OCTA is undeniably preferred for non-invasive evaluation of macular involvement. One study even suggests that OCTA could provide useful biomarkers for assessing macular perfusion in response to SCD systemic treatments [71].

Currently, there is no curative treatment of non-proliferative SCR. Most published studies recommend considering treatment beginning at Goldberg stage III, although supporting evidence remains limited and largely observational [29,72]. The main treatment option for proliferative SCR remains sectoral panretinal laser photocoagulation. It is generally used in cases of rapid neovascular growth, large neovessel lesions, bilateral involvement, VH or monocular status, especially if the contralateral eye has been lost due to SCR [73]. An alternative treatment option is the administration of anti-VEGF to reduce retinal neovascularization. However, this is not a long-term curative treatment, as it does not resolve retinal ischemia and carries a risk of recurrence [57,74].

Given the slow progression of SCR, with an estimated rate of 28.7% over 11 years [27], and the spontaneous regression of neovascularization that has been reported in about 61% of cases over 2 years [19,30], a conservative strategy of close monitoring is justified for small and isolated neovessels [14].

Long-term monitoring and individualized management are essential for optimizing outcomes in patients with sickle cell-related ocular disease [16,75]. We propose a preliminary scoring framework intended to generate hypotheses and guide future prospective validation. (Figure 5) A scoring system that integrates modifiable and non-modifiable risks to determine monitoring intervals of SCD patients.

Our findings are broadly consistent with previous reviews. Ribeiro et al. [75] and Abdalla Elsayed et al. [72] highlighted the importance of early detection and treatment of sickle cell retinopathy, but their studies were published before many recent advances in ultra-wide-field imaging and OCTA. Leitão Guerra et al. [68] demonstrated the value of OCTA for detecting early microvascular changes, a finding supported by our review. Similarly, our conclusions regarding treatment align with the Cochrane review by Myint et al. [73], which reported limited high-quality evidence for current therapeutic interventions despite their widespread clinical use.

Compared with these earlier reviews, our study incorporates more recent evidence, evaluates demographic and hematologic risk factors, and proposes a risk-adapted screening framework.

This systematic review has several strengths:

▪ Comprehensive search strategy across multiple databases with supplementary citation searching, identifying 48 eligible studies spanning 45 years of research.
▪ Validated quality assessment tools (Newcastle–Ottawa Scale for observational studies, Cochrane RoB 2 for RCTs) with transparent domain-level reporting.
▪ Geographic and population diversity with studies from 15 countries across 5 continents, enhancing generalizability.
▪ Clinically actionable outputs including risk stratification scores and management algorithms designed for implementation.
▪ PRISMA 2020 compliance with complete checklist reporting.

Several limitations should be acknowledged:

*Study-level limitations:*

- More than half of included studies were at moderate risk of bias, with common limitations including inadequate confounder adjustment, short follow-up, and single-center recruitment.
- The available RCTs were conducted in the 1980s–2000s with methodological limitations (open-label design, incomplete randomization description) that reduce confidence in treatment effect estimates.
- Substantial heterogeneity in outcome definitions, imaging protocols, and staging criteria precluded formal meta-analysis.

*Review-level limitations:*

- Study selection and quality assessment were performed by a single reviewer, which may have introduced selection bias despite the use of predefined eligibility criteria and standardized quality assessment tools.
- The Newcastle–Ottawa Scale, while widely used, has demonstrated fair-to-poor inter-rater reliability in methodological studies, and domain-level judgments involve subjective interpretation.
- Publication bias was not formally assessed due to the absence of meta-analysis; positive and statistically significant findings may be over-represented.
- The review was not prospectively registered in PROSPERO.
- The proposed risk stratification score and management algorithm are derived from evidence synthesis and have not been externally validated.

*Evidence gaps:*

- No RCTs compare screening strategies or intervals.
- Anti-VEGF therapy evidence is limited to small case series without controlled comparisons.
- Long-term visual acuity outcomes (>10 years) are poorly reported.
- Quality of life and patient-centered outcomes are rarely assessed.
- Cost-effectiveness data are absent.

Based on the identified evidence gaps, we suggest the following priorities for future research:

*For clinicians:*

- The proposed risk stratification framework should be prospectively validated in diverse populations before widespread implementation.
- Integration of wide-field imaging and OCTA into routine screening protocols should be evaluated for feasibility and cost-effectiveness in various healthcare settings.
- Collaborative care models between ophthalmology and hematology should be developed and evaluated.

*For researchers:*

- Randomized trials comparing screening strategies (modalities, intervals) are needed to establish evidence-based protocols.
- Controlled trials of anti-VEGF therapy, alone and in combination with laser, are warranted given the expanding use of these agents.
- Longitudinal studies with standardized outcome measures and extended follow-up (≥10 years) are essential to characterize disease natural history and treatment durability.
- Patient-reported outcome measures and quality of life assessments should be incorporated into future studies.
- Development of validated prognostic models incorporating clinical, hematological, and imaging biomarkers could enable precision medicine approaches with the goal of refining prognostic accuracy and optimizing personalized care.
- Investigation of novel disease-modifying therapies (gene therapy, emerging sickle cell treatments) and their effects on ocular outcomes represents an important frontier.

## 5. Conclusions

Risk-based management of SCR and SCM is feasible and necessary. This article proposes a protocol that can be applied by ophthalmologists to optimize patient outcomes. Given the high burden of care in SCD patients, ophthalmologic screening and treatment strategies should be tailored to minimize invasiveness while maximizing clinical benefit. Integrating patient risk factors and multimodal imaging results can help refine screening intervals and treatment thresholds. The recommendations made in this literature review provide a framework for improving the ophthalmological management of sickle cell patients. Further prospective studies are needed to validate these approaches and optimize long-term outcomes.

The proposed risk-adapted screening algorithm provides a pragmatic framework for individualized ophthalmologic follow-up. However, because it has not yet been externally validated, it should be considered hypothesis-generating and requires prospective validation before widespread clinical adoption.

## Figures and Tables

**Table 1:** List and scoring of the analyzed studies [15,16,17,18,19,20,21,22,23,24,25,26,27,28,29,30,31,32,33,34,35,36,37,38,39,40,41,42,43,44,45,46,47,48,49,50,51,52,53,54,55,56,57,58,59,60,61,62].

Authors	Year	Country	Type of Study	Number of Patients	Review	Content Brought in Relation to the Research Question	Score of Studies (CONSORT, STROBE)	Age (Years)
Duan et al. [15]	2019	United States of America	R	296	American Journal of Ophthalmology	RF	20/22	34.2 ± 12.1
Diallo et al. [16]	2009	Burkina-Faso	P	73	Journal Français d’Ophtalmologie	RF	20/22	21.4 ± 8.6
Dembélé et al. [17]	2017	Mali	P	1604	Rev Med Interne	RF	19/22	18.4 ± 11.2
Leveziel et al. [18]	2011	France	R	942	Medicine (Baltimore)	RF	19/22	28.7 ± 14.3
Downes et al. [19]	2005	Jamaica	R	473	Ophthalmology	E	20/22	Entry: 5–15
Fox et al. [20]	1990	Jamaica	P	1319	British Journal of Ophthalmology	RF	19/22	Entry: birth
Saidkasimova et al. [21]	2016	United Kingdom	P	182	European Journal of Ophthalmology	RF	20/22	32.1 ± 12.4
Rosenberg et al. [22]	2011	United States of America	R	108	Journal of the American Association for Pediatric Ophthalmology and Strabismus	RF	18/22	12.4 ± 4.2
Minniti et al. [23]	2025	United States of America	R	412	Eur J Haematol	RF	19/22	31.2 ± 15.4
Da Silva Costa et al. [24]	2024	Brazil	P	8	Exp Biol Med	RF	19/22	26.3 ± 10.1
Serjeant et al. [25]	1986	Jamaica, United-kingdom	P	27	Br J Ophthalmol.	RF	18/22	NR
Brandsen et al. [26]	2025	The Netherlands	P	68	Br J Haematol	RF	19/22	35.8 ± 14.1
Brandsen et al. [27]	2023	Netherlands	R	129	Blood Advances	RF	21/22	36.4 ± 13.8
Aleluia et al. [28]	2017	Brazil	P	200	BMC Hematology	RF	17/22	24.5 ± 15.2
Aguiar et al. [29]	2020	Brazil	R	97	Revista Brasileira de Oftalmologia	E	19/22	48.3 ± 7.1
Condon et al. [30]	1980	Jamaica	P	313	British Journal of Ophthalmology	E	21/22	NR
Bilong et al. [31]	2018	Cameroon	P	84	Journal Français d’Ophtalmologie	RF	21/22	22.3 ± 8.9
Hayes et al. [32]	1981	United Kingdom	P	261	Br J Ophthalmol.	RF	20/22	Adult
Mian et al. [33]	2018	United States of America	R	300	British Journal of Haematology	T	19/22	29.8 ± 11.6
Estepp et al. [34]	2017	United States of America	P	230	American Journal of Hematology	T	21/22	11.2 ± 4.8
Smith et al. [35]	2025	United States of America	R	630	Ophthalmology	T	20/22	Pediatric/adolescent
Fares et al. [36]	2021	France	P	118	Am J Ophthalmol	RF E	18/22	30.4 ± 11.8
Lim et al. [37]	2021	United States of America	P	370	JAMA Ophthalmology	RF E T	21/22	33.5 ± 14.2
Mathew et al. [38]	2015	United Kingdom	P	107	British Journal of Ophthalmology	E	20/22	31.2 ± 13.1
Orssaud et al. [39]	2023	France	R	132	Front Med	RF T	19/22	34.8 ± 12.7
Dell’Arti et al. [40]	2018	Italy	P	60	PLOS ONE	RF	21/22	29.3 ± 12.8
Li et al. [41]	2019	United States of America	R	398	Ophthalmology	RF E T	20/22	Pediatric
Babalola et al. [42]	2001	Nigeria	P	90	Afr J Med Med Sci	E	17/22	Pediatric
Gill et al. [43]	2001	Nigeria	R	263	Can J Ophthalmol	E	19/22	Pediatric
Han et al. [44]	2019	United States of America	P	35	RETINA	E T	17/22	31.8 ± 11.2
Alabduljalil et al. [45]	2021	Koweït	P	436	Br J Ophthalmol	E	18/22	28.4 ± 16.3
Spaide et al. [46]	2015	United States of America	P	12	JAMA Ophthalmol.	E	18/22	34.2 ± 9.5
Torres-Villaros et al. [47]	2022	France	P	44	J Clin Med	E	19/22	29.6 ± 10.8
Cho et al. [48]	2011	United States of America	R	6	RETINA	RF E	18/22	28.5 ± 8.3
Soni et al. [49]	2024	India	P	55	Pediatr Blood Cancer	E	21/22	12.3 ± 3.2
Hoang et al. [50]	2011	United States Of America	R	21	Am J Ophthalmol	E	17/22	33.5 ± 12.8
Enjalbert et al. [51]	2024	France	R	16	Retina	E	21/22	28.4 ± 9.8
Oruz et al. [52]	2025	Turkey	R	8	Turk J Ophthalmol	E	17/22	24.6 ± 8.2
Minvielle et al. [53]	2016	France	R	9	Am J Ophthalmol	E	20/22	32.1 ± 10.4
Jampol et al. [54]	1983	United States, Jamaica	RCT	167	Ophthalmology	T	19/25	NR
Farber et al. [55]	1991	United States of America	RCT	116	Arch Ophthalmol	T	17/25	NR
Sayag et al. [56]	2008	France	RCT	101	Eur J Ophthalmol.	T	24/25	28.5 ± 9.4
Okonkwo et al. [57]	2024	Nigeria	R	88	Eur J Ophthalmol	T	20/22	26.4 ± 9.8
Cai et al. [58]	2018	United States of America	R	5	J Vitreoretin Dis	T	17/22	35.2 ± 12.1
Ahmed et al. [59]	2024	United States of America	R	28	Retina	T	21/22	35.1 ± 14.2
Chen et al. [60]	2014	United States of America	R	14	Am J Ophthalmol	T	19/22	33.8 ± 11.5
Williamson et al. [61]	2009	United Kingdom	R	27	Eye Lond Engl	T	23/22	32.4 ± 10.2
Rohowetz et al. [62]	2024	United States of America	R	61	Ophthalmology	T	20/22	34.2 ± 13.5

R: retrospective study, P: prospective study, RCT: randomized controlled trial. RF: risk factors, E: examination, T: treatment.

**Table 2:** Overview of sickle cell retinopathy’s and sickle cell maculopathy’s risk factors [17,18,19,20,21,22,23,24,25,26,27,28,29,30,31,32,33,34,35,36,37,38,39,40].

Condition	Risk Level	Risk Factors
Sickle cell retinopathy	High	Genotype HbSC [15,16,17,18,19,20,21,22,23,24] Age > 30 [18,19,20,21,22,30,31,32] Male sex [17,19,20,29,32] Low fetal hemoglobin levels < 15% [17,27,33,34] Absence of hydroxyurea treatment [17,18,26]
	Moderate	High hemoglobin and hematocrit levels [17,25,26,27,28] High blood viscosity [26]
	Low	Systemic factors [31,32,33,34] No history of blood transfusion [26]
Sickle cell maculopathy	High	Genotype HbSS [36,37,38,39] Low fetal hemoglobin levels [40]
	Moderate	High hemoglobin and hematocrit levels [36] Systemic factors [40]
	Low	Prolonged prothrombin time [36] Elevated lactate dehydrogenase [36]

HbSC: hemoglobin SC, HbSS: hemoglobin SS. Classification proposed by the authors based on evidence synthesis; not externally validated.

## Data Availability

The data supporting the findings of this study are available within the article and its Supplementary Materials.

## Supplementary Materials

The following supporting information can be downloaded at: https://www.mdpi.com/article/10.3390/bioengineering13080867/s1, Table S1: PRISMA 2020 Checklist, Table S2: Risk-of-bias assessment for observational studies.

## Abbreviations

AAO	American Academy of Ophthalmology
Anti-VEGF	Anti-vascular endothelial growth factor
FA	Fluorescein angiography
FE	Fundus examination
HbF	Fetal hemoglobin
HbSC	Hemoglobin SC
HbSS	Hemoglobin SS
LDH	Lactate dehydrogenase
MCV	Mean corpuscular volume
OCTA	Optical coherence tomography angiography
PICO	Patient, intervention, comparison, outcome
PPV	Pars plana vitrectomy
PRISMA	Preferred Reporting Items for Systematic Reviews and Meta-Analyses
PT	Prothrombin time
RD	Retinal detachment
SCD	Sickle cell disease
SCM	Sickle cell maculopathy
SCR	Sickle cell retinopathy
SD-OCT	Spectral-domain optical coherence tomography
UWFA	Ultra-wide-field fluorescein angiography
UWFP	Ultra-wide-field fundus photography
VEGF	Vascular endothelial growth factor
VH	Vitreous hemorrhage
WFA	Wide-field fluorescein angiography
