# Supplementary material for: Retinal Challenges in Sickle Cell Patients—Present and Future: A Systematic Review
Source: Bioengineering (Basel). 2026 Jul 27;13(8):867. doi: 10.3390/bioengineering13080867 (PMC13509248; doi:10.3390/bioengineering13080867)
Supplement: Supplementary file 1 [file bioengineering-13-00867-s001.zip › bioengineering-4395671-supplementary.pdf]

Table S1. PRISMA 2020 Checklist.

Section and Topic	Item #	Checklist Item	Location Where Item Is Reported
**Title**			
Title	1	Identify the report as a systematic review.	1
Abstract			
Abstract	2	See the PRISMA 2020 for Abstracts checklist.	1
Introduction			
Rationale	3	Describe the rationale for the review in the context of existing knowledge.	2
Objectives	4	Provide an explicit statement of the objective(s) or question(s) the review addresses.	1–2
Methods			
Eligibility criteria	5	Specify the inclusion and exclusion criteria for the review and how studies were grouped for the syntheses.	3
Information sources	6	Specify all databases, registers, websites, organisations, reference lists and other sources searched or consulted to identify studies. Specify the date when each source was last searched or consulted.	3
Search strategy	7	Present the full search strategies for all databases, registers and websites, including any filters and limits used.	3–4
Selection process	8	Specify the methods used to decide whether a study met the inclusion criteria of the review, including how many reviewers screened each record and each report retrieved, whether they worked independently, and if applicable, details of automation tools used in the process.	3
Data collection process	9	Specify the methods used to collect data from reports, including how many reviewers collected data from each report, whether they worked independently, any processes for obtaining or confirming data from study investigators, and if applicable, details of automation tools used in the process.	3
Data items	10a	List and define all outcomes for which data were sought. Specify whether all results that were compatible with each outcome domain in each study were sought (e.g., for all measures, time points, analyses), and if not, the methods used to decide which results to collect.	3
	10b	List and define all other variables for which data were sought (e.g., participant and intervention characteristics, funding sources). Describe any assumptions made about any missing or unclear information.	3
Study risk of bias assessment	11	Specify the methods used to assess risk of bias in the included studies, including details of the tool(s) used, how many reviewers assessed each study and whether they worked independently, and if applicable, details of automation tools used in the process.	3
Effect measures	12	Specify for each outcome the effect measure(s) (e.g., risk ratio, mean difference) used in the synthesis or presentation of results.	8

Synthesis methods	13a	Describe the processes used to decide which studies were eligible for each synthesis (e.g., tabulating the study intervention characteristics and comparing against the planned groups for each synthesis (item #5)).	4
	13b	Describe any methods required to prepare the data for presentation or synthesis, such as handling of missing summary statistics, or data conversions.	4
	13c	Describe any methods used to tabulate or visually display results of individual studies and syntheses.	5–14
	13d	Describe any methods used to synthesize results and provide a rationale for the choice(s). If meta-analysis was performed, describe the model(s), method(s) to identify the presence and extent of statistical heterogeneity, and software package(s) used.	4
	13e	Describe any methods used to explore possible causes of heterogeneity among study results (e.g., subgroup analysis, meta-regression).	NA (narrative synthesis)
	13f	Describe any sensitivity analyses conducted to assess robustness of the synthesized results.	NA
Reporting bias assessment	14	Describe any methods used to assess risk of bias due to missing results in a synthesis (arising from reporting biases).	3
Certainty assessment	15	Describe any methods used to assess certainty (or confidence) in the body of evidence for an outcome.	5
Results			
Study selection	16a	Describe the results of the search and selection process, from the number of records identified in the search to the number of studies included in the review, ideally using a flow diagram.	5
	16b	Cite studies that might appear to meet the inclusion criteria, but which were excluded, and explain why they were excluded.	3
Study characteristics	17	Cite each included study and present its characteristics.	5–8
Risk of bias in studies	18	Present assessments of risk of bias for each included study.	5–8
Results of individual studies	19	For all outcomes, present, for each study: (a) summary statistics for each group (where appropriate) and (b) an effect estimate and its precision (e.g., confidence/credible interval), ideally using structured tables or plots.	8–14
Results of syntheses	20a	For each synthesis, briefly summarise the characteristics and risk of bias among contributing studies.	5–14
	20b	Present results of all statistical syntheses conducted. If meta-analysis was done, present for each the summary estimate and its precision (e.g., confidence/credible interval) and measures of statistical heterogeneity. If comparing groups, describe the direction of the effect.	8–14

	20c	Present results of all investigations of possible causes of heterogeneity among study results.	18
	20d	Present results of all sensitivity analyses conducted to assess the robustness of the synthesized results.	NA
Reporting biases	21	Present assessments of risk of bias due to missing results (arising from reporting biases) for each synthesis assessed.	NA
Certainty of evidence	22	Present assessments of certainty (or confidence) in the body of evidence for each outcome assessed.	NA
Discussion			
Discussion	23a	Provide a general interpretation of the results in the context of other evidence.	14–15
	23b	Discuss any limitations of the evidence included in the review.	15
	23c	Discuss any limitations of the review processes used.	15
	23d	Discuss implications of the results for practice, policy, and future research.	15–16
Other information			
Registration and protocol	24a	Provide registration information for the review, including register name and registration number, or state that the review was not registered.	NA
	24b	Indicate where the review protocol can be accessed, or state that a protocol was not prepared.	NA
	24c	Describe and explain any amendments to information provided at registration or in the protocol.	NA
Support	25	Describe sources of financial or non-financial support for the review, and the role of the funders or sponsors in the review.	16
Competing interests	26	Declare any competing interests of review authors.	16
Availability of data, code and other materials	27	Report which of the following are publicly available and where they can be found: template data collection forms; data extracted from included studies; data used for all analyses; analytic code; any other materials used in the review.	16

Table S2. Risk-of-bias assessment for observational studies.[15-43,45-63]

Authors	Assessment Tool	Overall Risk of Bias	Main Methodological Concerns
Duan et al. [15]	NOS	Moderate	Single-center study; cross-sectional design
Diallo et al. [16]	NOS	Moderate	Limited sample; single-center in Sub-Saharan Africa
Dembélé et al. [17]	NOS	Moderate	Small sample size; limited adjustment for confounders
Leveziel et al. [18]	NOS	Moderate	Single-center recruitment

Downes et al. [19]	NOS	Low	Limited external validity
Fox et al. [20]	NOS	Low	Long follow-up; limited covariate adjustment
Saidkasimova et al. [21]	NOS	Moderate	Single-center; heterogeneous population
Rosenberg et al. [22]	NOS	Moderate	Retrospective; pediatric population only
Minniti et al. [23]	NOS	Moderate	Retrospective; healthcare utilization data
Da Silva Costa et al. [24]	NOS	High	Very small sample ; molecular focus
Serjeant et al. [25]	NOS	Moderate	Historical cohort; older laboratory methods
Brandsen et al. [26]	NOS	Low	Prospective; hemorheological focus
Brandsen et al. [27].	NOS	Low	Moderate loss to follow-up
Aleluia et al. [28]	NOS	Moderate	Cross-sectional design; small sample size
Aguiar et al. [29]	NOS	Moderate	Retrospective design; selection bias possible
Condon et al. [30]	NOS	Low	Historical data; older imaging modalities
Bilong et al. [31]	NOS	Low	Small sample; limited generalizability
Hayes et al. [32]	NOS	Low	Prospective; older hematological assays
Mian et al. [33]	NOS	Moderate	Retrospective design; potential confounding
Estepp et al. [34]	NOS	Low	Single-center; selection bias possible
Smith et al. [35]	NOS	Low	Large cohort; retrospective design
Fares et al. [36]	NOS	Low	Prospective OCTA study; comprehensive risk factor analysis
Lim et al. [37]	NOS	Low	Prospective design; moderate sample size
Mathew et al. [38]	NOS	Low	Small sample; limited follow-up duration
Orssaud et al. [39]	NOS	Moderate	Retrospective; incomplete data for some variables
Dell'Arti et al. [40]	NOS	Low	Moderate sample; comprehensive OCT analysis
Li et al. [41]	NOS	Low	Retrospective; single institution
Babalola et al. [42]	NOS	Moderate	Older study; limited imaging technology available
Gill et al. [43]	NOS	Moderate	Retrospective; limited imaging modalities
Han et al. [45]	NOS	Moderate	Small sample; limited statistical power
Alabduljalil et al. [46]	NOS	Moderate	Cross-sectional comparison; operator variability
Spaide et al. [47]	NOS	High	Very small sample ; exploratory study
Torres-Villaros et al. [48]	NOS	Moderate	Small sample size; single-center
Cho et al. [49]	NOS	High	Very small sample ; case series
Soni et al. [50]	NOS	Low	Prospective; pediatric population

Hoang et al. [51]	NOS	Moderate	Small sample; retrospective OCT analysis
Enjalbert et al. [52]	NOS	Moderate	Small sample ; longitudinal OCTA data
Oruz et al. [53]	NOS	High	Very small sample; limited generalizability
Minvielle et al. [54]	NOS	Moderate	Small sample; retrospective design
Farber et al. [55]	RoB 2	Moderate	Randomization concerns; incomplete follow-up
Jampol et al. [56]	RoB 2	Moderate	Open-label design; some attrition
Sayag et al. [57]	RoB 2	Low	Prospective RCT; adequate randomization
Cai et al. [58]	NOS	High	Very small sample ; case series
Okonkwo et al. [59]	NOS	Moderate	Retrospective; single-center in resource-limited setting
Ahmed et al. [60]	NOS	Moderate	Small sample; surgical case series
Chen et al. [61]	NOS	Moderate	Small sample; retrospective design
Williamson et al. [62]	NOS	Moderate	Small sample; variable follow-up duration
Rohowetz et al. [63]	NOS	Moderate	Retrospective; surgical outcomes only

RoB 2: Cochrane Risk of Bias 2, NOS: Newcastle–Ottawa Scale.
